# Commentary: Transarterial Embolization in Neonatal Kasabach–Merritt Syndrome

**DOI:** 10.3389/fped.2022.846207

**Published:** 2022-06-20

**Authors:** Huaijie Wang, Zhengtuan Guo

**Affiliations:** Department of Pediatric Surgery, Xi'an International Medical Center Hospital, Xi'an, China

**Keywords:** Kaposiform hemangioendothelioma, tufted angioma, Kasabach-Merritt phenomenon, hemangioma, sirolimus, embolization, vascular anomalies

## Introduction

In the article entitled “Transarterial Embolization in Neonatal Kasabach-Merritt Syndrome” Wang et al. reported eleven neonates with Kasabach-Merritt phenomenon (KMP) treated with transarterial embolization (TAE) (1). We compliment the authors for their excellent results. Nevertheless, we have a few comments on the diagnosis and management of KMP.

## Comments and Discussion

### Confusion in Terminology and Definition Regarding KMP

The authors defined KMP as giant hemangioma - thrombocytopenia syndrome, which is outdated. In 1997, Sarkar et al. revealed KMP was associated with Kaposiform hemangioendothelioma (KHE) and not with common infantile hemangioma (2). In the same year, Enjolras et al. found infants with KMP did not have “true” classical hemangiomas (3). Currently, KMP is defined as a coagulopathy (thrombocytopenia) associated with KHE and tufted angioma (TA) (4). KHE and TA are considered to be synonymous vascular tumors with similar presenting symptoms and potential for KMP (5). Thus, KMP specifically occurs in KHE and TA, does not occur with common hemangioma.

Terminologies associated with KMP used in this report included hemangioma, Kaposi hemangioendothelioma and tufted hemangioma. In the latest ISSVA (the International Society for the Study of Vascular Anomalies) classification for vascular anomalies, the term ***hemangioma*** encompass a broad group of tumors, including infantile hemangioma, congenital hemangioma, spindle-cell hemangioma, epithelioid hemangioma, hobnail hemangioma, etc. (5). As we mentioned above, KMP occurs exclusively with KHE and TA. Incorrect use of the term ***hemangioma*** in KMP adds to the confusion regarding the diagnosis, understanding and management of this entity, which should be avoided.

Terms ***Kaposi hemangioendothelioma*** and ***tufted hemangioma*** are also incorrect and should be abandoned. In the latest ISSVA classification for vascular anomalies, correct nomenclatures are ***Kaposiform hemangioendothelioma*** and ***tufted angioma*** (5).

### KMP Does Not Occur in Hepatic Congenital Hemangioma

In the 11 neonates reported, we noticed three had hepatic hemangioma. All three hemangiomas are unifocal, which suggests the diagnosis of congenital hemangioma (CH) rather than KHE (6). KHE or TA has never been confirmed and found in liver parenchyma in literature (6).

CH proliferates *in utero* and reaches peak size just prior to or at birth. Diagnosis may be made prenatally. Possible complications with hepatic CH include intralesional bleeding, thrombocytopenia, hypofibrinogenemia, and high-output cardiac failure (6). However, thrombocytopenia and hypofibrinogenemia associated with CH is not KMP. These laboratory findings in CH are generally transient, mild to moderate, and rarely necessitate interventions, which can improved naturally as CH regresses spontaneously. Also, we noticed high output heart failure developed in one patient (maybe the case 6 with a hepatic hemangioma). This is a typical complication with hepatic CH and is not a known feature of KMP. High output heart failure has never been found in confirmed KHE in literature.

Additionally, images provided in the Figure 2 of article strongly support the diagnosis of hepatic CH. Typically, hepatic CH shows to be a well-defined, solitary, spherical tumor with centripetal enhancement and central sparing on computed tomography or enhanced magnetic resonance imaging (6). Images in the Figures 2C,E showed typical features above.

### Sirolimus Shows Very Promising Results for KMP

Medical treatment is the mainstay in KMP management, mainly including vincristine, corticosteroids and sirolimus. These drugs can be administered as the first-line treatment (7, 8). Especially, sirolimus also has shown very excellent response for multimodal-resistant KHE/KMP (7). Until now, no response to sirolimus has never been reported in confirmed KHE/KMP in literature.

Authors used single corticosteroids as the initial therapy. However, the overall response rate of this monotherapy is relatively low, ranging 10–27% (7). The continuing propranolol was administered in four patients. For managing vascular anomalies, infantile hemangioma is considered to be the only entity responsive to propranolol. No study has confirmed therapeutic role of propranolol in KHE/KMP. The statement “Glucocorticoids, sirolimus, or propranolol is most commonly used to treat KMS” is inappropriate. Propranolol is not used to treat KHE/KMP.

Additionally, the very short duration of treatment (3–10 days) may contribute to the “poor effect” of medication prior to TAE. Even the most promised sirolimus administering, the time of platelet count renormalization is still more than a week (8).

Consequently, sirolimus alone or sirolimus based combination therapy (plus steroids and/or vincristine) is recommended as a first-line therapy for the treatment of KHE/KMP.

### TAE Plays an Additive Role to Systemic Treatment for KMP

The additive role of TAE to systemic sirolimus for KMP has been reported recently, in which adjunct TAE induced a more rapid resolution of KMP, however, tumor response as well as rebound rates were similar to the group of sirolimus only (9). TAE have historically been used in KHE. The effects have proven to be temporary and are currently used primarily as a temporizing role to provide additional time for medical therapy to be effective, or as an adjunct to surgery to reduce bleeding during a planned surgery (4).

In literature, complete response to TAE only has not been reported in KHE/KMP. All had at least two modalities of treatment regarding TAE for KHE/KMP (10). TAE showed no response or temporary partial response in literature (4, 10). TAE should not be considered as the main treatment for KHE/KMP.

Authors did not provided data of follow-up. Considering the temporary effect and adjunctive role of TAE to systemic medication for KHE/KMP, the follow-up is very important for readers.

## Author Contributions

HW conceptualized and designed this study, and drafted the manuscript. ZG contributed to literature research and manuscript review. All authors contributed to the article and approved the submitted version.

## Conflict of Interest

The authors declare that the research was conducted in the absence of any commercial or financial relationships that could be construed as a potential conflict of interest.

## Publisher's Note

All claims expressed in this article are solely those of the authors and do not necessarily represent those of their affiliated organizations, or those of the publisher, the editors and the reviewers. Any product that may be evaluated in this article, or claim that may be made by its manufacturer, is not guaranteed or endorsed by the publisher.
